# The Effect of Muscle Stretching on Joint Position Sense in Active Young and Elderly Adults: A Comparative Study

**DOI:** 10.3390/muscles3030025

**Published:** 2024-08-23

**Authors:** Thomas Haab, Peter Leinen, Madeleine Stanek

**Affiliations:** 1 Sport Science Institute, Saarland University, 66123 Saarbrücken, Germany; p.leinen@mx.uni-saarland.de (P.L.); madeleine.stanek1@web.de (M.S.); 2 IFAA Hochschule GmbH, 68775 Ketsch, Germany

**Keywords:** proprioception, muscle stretching, age, joint position sense

## Abstract

Previous studies have revealed decreased proprioception and perception of joint position in elderly adults. Joint position sense, indicating an individual’s ability to perceive the position of limbs without visual aid, is essential for everyday movements. A few studies have shown a positive effect of muscle stretching on joint position sense (JPS). However, these studies have only been conducted with younger participants. The impact of stretching on JPS in elderly adults remains unclear. Therefore, the aim of this study is to compare the acute effects of muscle stretching on joint position sense in young and elderly adults. An experimental group of younger adults (*n* = 15; 25.4 ± 2.9 years) and an experimental group of active, healthy elderly adults (*n* = 15; 64 ± 3.5 years) stretched their knee flexor muscles. The absolute error (AE) of the joint position sense was calculated before and after muscle stretching. The results indicated no significant difference in the AE between groups (*p* > 0.05) after the stretching intervention. The similarity in the physical activity status of the subjects may have influenced the results. Even though no significant age-specific differences were found in this study, its results may not be applicable to non-active elderly adults.

## 1. Introduction

The ability to recognize the position of joints is a fundamental part of proprioception, a sensory system that plays a central role in the performance of everyday activities. These activities range from walking to sporting activities. This sensory capability not only underpins the detection and modulation of bodily motions, but also ensures the individual’s spatial awareness, overall coordination, and efficient joint operation. Notably, research has highlighted a decrease in proprioception among older individuals [1]. This finding underlines the importance of understanding the mechanisms underpinning this decline. In the context of this study, proprioception is conceptualized, following the definition provided by Héroux et al. [2], as the awareness of the body’s mechanical and spatial states, encapsulating the structural and functional aspects of its musculoskeletal system. Such a definition provides the possibility to consider the existence of different dimensions of the sense of position: the body’s own perception of limb position and the positioning of the limbs in the broader environmental context. This classification suggests a layered approach to proprioception, where low-level proprioception is concerned with a singular, straightforward frame of reference, and high-level proprioception embraces a more complex, composite frame of reference. Within the spectrum of low-level proprioception lies the concept of joint position sense (JPS), which describes the ability to discern the spatial orientation of a limb without the aid of visual input [3]. Empirical evidence from a prior investigation indicates that muscle stretching exercises yield positive outcomes on JPS among younger cohorts [4]; however, the effects of such interventions on the JPS capabilities of the elderly population have yet to be fully elucidated. This gap in knowledge invites further research on how proprioceptive abilities, especially in relation to joint position sensing, evolve with age and how they can be maintained or enhanced through specific physical activities or therapeutic strategies.

Over the course of a lifetime, flexibility is estimated to decrease by around 50% [5]. This decrease is particularly pronounced in the elderly population, who often exhibit a notably reduced range of motion (ROM) [6,7]. Given this decline, stretching exercises have been strongly recommended for the elderly to improve their flexibility [8]. Despite the commonly observed reduction in ROM among older individuals, studies have shown that they are capable of improving their flexibility through acute stretching protocols as effectively as their younger counterparts [9]. This suggests that the enhancement of one’s flexibility through stretching is not significantly compromised by age. Another study that conducted a standardized stretching program over 10 weeks supports this result. It revealed no substantial differences in the extent of ROM improvement between young and elderly participants [10]. These findings challenge prevailing assumptions about the inevitability of declining physical capabilities with age. They further highlight the potential for targeted interventions to mitigate some of the adverse effects associated with aging on flexibility and mobility.

The observed reduction in ROM in elderly adults can be partially explained by several age-related changes within the muscular system. A significant decline in muscle mass can be noted, which is further characterized by reductions in the size and number of muscle fibers [11,12,13]. This decrease in muscle mass and fiber alterations are critical factors that contribute to the decline in physical capabilities observed in older populations. Additionally, the aging process is associated with an increased accumulation of connective tissue within the muscles. This augmented presence of connective tissue leads to enhanced muscle stiffness [14], intensifying the reduction in ROM. These physiological changes highlight the complex interplay between muscle composition, connective tissue proliferation, and their collective impact on the flexibility and, overall, the mobility of elderly individuals.

The alteration of flexibility that accompanies aging is parallel with changes in proprioception and, therefore, in JPS [1,15]. The current consensus within the scientific community, as outlined by Héroux et al. in 2022 [2], posits that proprioception is mediated by an intricate interplay of sensory inputs from muscle receptors, joint receptors, and Golgi tendon organs across all joints. The debate about the specific contributions of these receptor types to proprioception remains vibrant. Studies by Burgess et al. [16], McCloskey [17], and Proske et al. [18] emphasize the role of muscle receptors in the proprioceptive process. This perspective is further explored by Proske and Gandevia [15], who discussed the empirical challenges of conclusively determining the role of joint receptors in sensory feedback, given the methodological hurdles in isolating these receptors for independent analysis. Furthermore, they present a compelling query regarding the mechanisms enabling patients with artificial hip joints, who lack the natural capsules and ligaments, to effectively use their sense of limb position and movement immediately after surgery. Proske et al. [19] discovered that muscle spindles exhibit thixotropic properties, which means that the responsiveness of the intrafusal fibers within these spindles is influenced by the history of muscle contractions and changes in muscle length. This characteristic suggests a dynamic interaction whereby previous muscular activities can significantly affect the spindle’s sensitivity to changes in limb position and movement [20]. Thus, it is inferred that stretching exercises that precede proprioceptive engagement could have a modulatory effect on JPS. This occurs primarily through the adjustment of afferent signals emanating from muscle spindles, highlighting the potential for proactive interventions to enhance proprioceptive accuracy through targeted physical activities.

With increasing age, there is a notable decline in the coordination and accuracy of motor movements, which significantly impacts stability, balance, and the control of limb movements. Hence, the capacity for proprioceptive perception, which is critical for the body’s ability to perceive and respond to its position and movement in space, diminishes. Ferlinc et al. [1] emphasize the critical role of continuous physical mobility in reducing falls among the elderly. Petrella et al. [21] conducted a comparative study of young and elderly subjects across various levels of physical fitness, discovering that physical activity can mitigate the decline in JPS associated with aging. This research underscores the potential of physical exercise to enhance proprioceptive function and suggests that it could play a crucial role in preventing falls and promoting the overall well-being of the elderly.

Various studies, using different methods to assess the effects of aging on proprioception and JPS, have reported a wide range of results, some of which appear to contradict each other. A substantial portion of this research supports the assertion that elderly individuals experience notable deficits in proprioceptive abilities when compared to their younger counterparts. However, specific studies, like the one by Djajadikarta et al. [20], could not decisively confirm a decline in these sensory functions directly correlated with aging. Schmidt et al. [22] designed an experiment where participants’ arms were passively positioned at specific angles, and they were then asked to verbally confirm when these angles were reached without the aid of visual cues. Interestingly, this study did not identify any significant discrepancies in proprioceptive accuracy across different age groups. However, Petrella et al. [21] investigated proprioception by asking participants to adjust their legs to predefined angles from a starting neutral position repeatedly. Their findings pointed towards an age-related decline in proprioceptive accuracy. These varied outcomes highlight the complexity of understanding proprioceptive changes with aging. The diversity of findings underscores the need for further nuanced research to unravel these elements and better understand the mechanisms behind proprioceptive changes in the elderly.

The theory that stretching could have a positive effect on JPS has been the subject of various studies, although the results have not been consistent. Studies by Larsen et al. [23], Moradi et al. [24], and Torres et al. [25] suggest that static stretching does not significantly alter JPS. In contrast, findings by Ghaffarinejad et al. [4] indicate a positive impact of stretching on JPS, albeit limited to specific joint angles and muscle groups. Further support for the potential benefits of stretching on JPS is provided by research from Pradeep et al. [26] and Walsh [27], which points to a beneficial effect, contributing to the mixed evidence in this area. One notable limitation of these research studies is the focus on younger participants, typically aged between 20 and 30 years, leaving a considerable gap in our understanding of how stretching influences JPS among older adults. This oversight underscores the necessity for targeted research with the aim of exploring the effects of stretching exercises on proprioceptive accuracy within the elderly population.

Given the previously documented age-specific responses to muscle stretching interventions [10,28], the aim of this study was to investigate the hypothesis that an acute stretching intervention affects the JPS of older adults in the same way as in younger adults. If neuromuscular function declines with age, it could be hypothesized that elderly individuals might exhibit a greater absolute error in the JPS measurements compared to their younger counterparts.

## 2. Results

**Figure 1:**
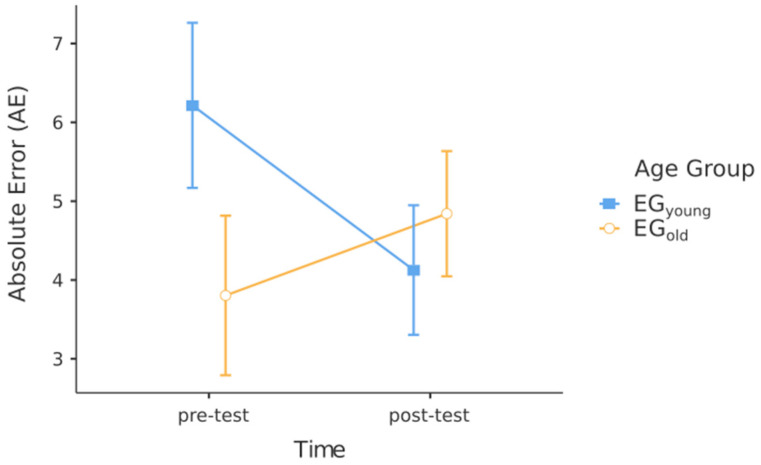
Mean and SEM of AE.

The results of the absolute error (AE) analysis revealed a group x time interaction (F(1, 28) = 4.41, *p* < 0.05, η² = 0.14) (Figure 1). However, we found no significant main effect of time (F(1, 28) = 0.47, *p* = 0.50) and no significant main effect of group (F(1, 28) = 0.36, *p* = 0.56). Pairwise comparisons indicated no significant differences between pre- and post-tests in the young experimental group (EG_young_) (pre-test: M = 5.98, SEM = 1.0 vs. post-test: M = 3.93, SEM = 0.79; *p* = 0.059) and the elderly experimental group (EG_old_) (pre-test: M = 3.80, SEM = 1.0 vs. post-test: M = 4.84, SEM = 0.79; *p* = 0.38).

The EG_young_ displayed higher but non-significant AE values in the pre-test compared to the post-test. Contrary to our second hypothesis, the EG_old_ had lower, albeit non-significant, AE values in the pre-test compared to the EG_young_. We assumed that the EG_old_ would demonstrate a higher AE due to a decline in neuromuscular function and reduced receptor sensitivity. Interestingly, the EG_old_ exhibited an increase in AE from pre- to post-test, while the EG_young_ showed a decrease in AE.

## 3. Discussion

The aim of our study was to evaluate the acute impact of muscle stretching on knee joint position sense (KJPS) across young and elderly adults. This research aimed to ascertain whether the effectiveness of muscle stretching in modulating the KJPS exhibits any variance between these demographic groups. Based on the existing literature that outlines age-related declines in neuromuscular function and proprioceptive accuracy, our study was driven by two hypotheses: (1) an acute muscle stretching in older adults has a comparable effect on KJPS as in younger adults; and (2) the aging process, characterized by a decrease in neuromuscular capabilities, manifests a heightened absolute error (AE) in the KJPS measurements during the pre-test phase among elderly participants compared to their younger counterparts. This approach aims not only to illuminate the immediate results of stretching exercises on proprioceptive function, but also to explore the nuanced dynamics of aging and neuromuscular health, particularly in the context of proprioceptive accuracy and joint sensory feedback mechanisms. Regarding our results, the AE showed a significant interaction between age group and pre- and post-test (*p* < 0.05). However, pairwise comparisons revealed no significant differences either between or within the groups. This aligns with previous studies [23,25], which have reported that acute stretching does not significantly influence JPS.

Ferlinc et al. [1] observed a marked deterioration in proprioception, attributing this decline to diminished biomechanical efficiency and neuromuscular control. This observation is also confirmed by Lee and Lim [29], who identified a direct negative relationship between advancing age and the accuracy of knee joint position sense (KJPS), highlighting the impact of aging on proprioceptive abilities. Proske and Gandevia [15] draw attention to sarcopenia, the age-related loss of muscle mass and function, as a significant factor contributing to the weakening of proprioceptive capabilities. Furthermore, aging is associated with changes in muscle spindle receptors and reduced efficiency in the neuromuscular transmission of stimuli. The aging process leads to a decline in the number and responsiveness of motor units, further impairing proprioceptive feedback. This reduction in proprioceptive perception plays a critical role in undermining an individual’s ability to maintain stability and balance, thereby significantly elevating the risk of falls among the elderly population. Falls in this demographic group are of substantial concern due to their association with severe injuries and the consequent impact on health and quality of life. Such changes in proprioceptive perception could potentially be influenced by the response of the muscles to stretching by increasing the responsiveness of the receptors and improving the transmission of information about joint angles to the central nervous system. This phenomenon has been investigated with mixed results [4,23,24,25,26,27]. Given these findings, the role of regular physical activity and targeted proprioceptive training emerges as a conceivable strategy to sustain mobility and reduce the incidence of falls in older adults, warranting further investigation.

A comparative analysis of various studies investigating the relationship between muscle stretching and JPS presents a dichotomy. Research conducted by Ghaffarinejad et al. [4], Pradeep et al. [26], and Walsh [27] suggests a beneficial effect of stretching exercises on JPS, offering evidence that such physical interventions can enhance proprioceptive accuracy. Conversely, studies by Larsen et al. [23], Moradi et al. [24], and Torres et al. [25] offer contradictory results, indicating no significant influence of stretching on JPS accuracy. These disparities underline the complexity of the interaction between muscle stretching, proprioceptive function, and the influence of different stretching protocols. The study by Larsen et al. [23], which utilized a specific stretching protocol involving three 30 s stretches, provides valuable insights into the nuanced effects of stretching on the ability of young adults to accurately perceive their joint positions. However, it is critical to recognize that the results of this study are inherently tied to the unique stretching program and cannot necessarily be extrapolated to all forms of muscle stretching or their effects on JPS in different populations or durations of stretching.

In our study, we replicated the stretching protocol used by Larsen et al. [23] and, similarly, observed non-significant differences in the AE values of the KJPS after the stretching intervention. Notably, our findings for the elderly group (EG_old_) indicated a reversal in the expected trend, with a higher KJPS accuracy observed in the pre-test compared to the post-test. This observation suggests that the stretching intervention may have led to a diminished sensitivity of proprioceptors in detecting knee joint position among elderly participants, in contrast to the young group (EG_young_), where stretching appeared to enhance proprioceptor sensitivity. This divergence in outcomes highlights the potential of age-related differences in response to stretching interventions on proprioceptive sensitivity. It underscores the necessity of further research to explore how varying stretching routines and durations might differently affect proprioceptive accuracy among diverse age groups, and to identify the underlying mechanisms that contribute to these varied responses.

The enhancement of range of motion (ROM) through stretching exercises in the aging population is a well-documented phenomenon, supported by numerous studies [10,28,30]. Despite this established benefit, the impact of stretching on JPS within this demographic group seems to be minimal. The prevailing literature indicates that proprioception experiences a decline in elderly individuals due to changes in muscle spindle receptors, muscle composition, and deficits in the neuromuscular system’s signal capabilities [15]. It was anticipated that these factors would result in older participants demonstrating a greater AE when compared to their younger counterparts. Counterintuitively, the initial comparison of the AE values between the young and elderly participants revealed that the younger individuals had higher AE values. This unexpected result might be explained by the possibility that older adults leverage their accumulated life-long experiential knowledge about limb positioning, enabling them to more accurately replicate specified angles. This finding introduces a perspective that contrasts with the hypothesis proposed by Stelmach and Sirica [31]. They suggest that the observed reduction in proprioceptive precision may not solely be an attribute of the proprioceptive system’s degeneration but might also involve the cognitive processing, or “recollection”, of proprioceptive information. Their approach involved a rematch test, wherein participants were asked to mirror the position of one arm with the other, relying exclusively on proprioceptive cues in the absence of visual input. This experiment highlighted a trend wherein older participants exhibited increased errors in replicating larger angles, in contrast to the relatively stable error rates among younger individuals. This extended explanation for the reduced precision in performance is an interesting point that should not go unnoticed in the discussion on this topic, even if our results do not directly support the assumption. However, the influence of cognitive factors could be a possible explanation for the varied results in this field and should be addressed in more detail in future studies. Following the intervention in our study, younger participants (EG_young_) demonstrated slightly better outcomes in terms of AE compared to their older counterparts (EG_old_), while the older group showed a slight increase in AE. Although these differences were not statistically significant, they indicate possible trends. The notion that older adults might exhibit deteriorated performance post-stretching, attributed to diminished receptor function and compromised signal transmission, cannot be conclusively validated based on our findings; nevertheless, the observed trends offer some support for this theory. An alternative explanation for the varied outcomes after stretching could be the thixotropic nature of muscle components, suggesting that muscle sensitivity to muscle length changes and contraction patterns may influence proprioceptive feedback [20]. To further validate these observations and to understand the nuanced effects of aging on proprioceptive accuracy after stretching exercises, additional research is necessary. Future studies should aim to compare JPS between younger and older adults more comprehensively, thereby shedding light on the aging process’s impact on proprioceptive accuracy and examining the potential role of thixotropic changes in muscle tissue on proprioceptive feedback mechanisms.

The findings of this study contribute to the growing body of research on the relationship between muscle stretching and JPS. Our results indicate that the effectiveness of muscle stretching on JPS may vary across different age groups, as we observed opposing trends between young and elderly adults. Specifically, the stretching regime implemented in this study did not result in significant improvements in the KJPS for the elderly group. It is important to recognize the limitations of our study, including its focus on a specific stretching program and the absence of an assessment of various stretching methods, intensities and durations. Future research should investigate these aspects to gain a more comprehensive understanding of how muscle stretching affects JPS in both young and elderly adults. Moreover, while similarities in JPS between young experimental and control groups were noted in other studies [23,25], it is unclear if these similarities hold in older populations, where age-related sensorimotor changes could influence proprioceptive abilities. This underscores the need to consider age-related differences in responses to muscle stretching. Notably, muscle fatigue, which may be influenced by factors such as quadriceps endurance, has been suggested to negatively affect JPS [32]. Therefore, elderly individuals, who are more prone to muscle fatigue, might respond differently to static stretching in terms of JPS. An additional consideration in our study is that all participants were active in sports. A comparative study by Venâncio et al. [33], involving roller hockey players and non-athletes, found that KJPS was higher in the roller hockey players. This suggests that proprioceptive perception may be enhanced in physically active individuals, as were the participants in our study. More specifically, Petrella et al. [21] have shown that physical activity has the potential to reduce the decline in the natural degeneration of JPS with age. This factor may also have influenced our results, which shows how important it is to consider the participants’ physical activity levels, sports practiced, and hours of exercise per week in future studies on this topic.

## 4. Materials and Methods

This study involved 30 participants divided into young (EG_young_ = 24.5 ± 2.9 years, BMI 24.76 ± 3.73 kg/m^2^) and elderly (EG_old_ = 64.0 ± 3.5 years, BMI 25.92 ± 2.93 kg/m^2^) experimental groups. Participants were involved in recreational sports for 3 to 5 h weekly. None of the participants had known neurological, cognitive, rheumatic, or orthopedic diseases, nor did they report any pain or injuries in their lower extremities. The required sample size for this study was estimated based on the methodologies used in previous studies by Ghaffarinejad et al. [4] and Larsen et al. [23]. All participants provided written informed consent prior to their inclusion in this study. Additionally, they indicated their dominant leg through a self-report questionnaire. This study was conducted in accordance with the ethical standards of the Declaration of Helsinki and was reviewed and approved by the Ethics Committee of Saarland University.

A quasi-experimental design was employed to assess the knee joint positioning sense (KJPS) of the dominant leg. The experimental procedure, as outlined in Table 1, involved three steps: (1) preparation, which included setting up the knee joint angular measurement apparatus and conducting a pre-test to evaluate each participant’s ability to perceive their KJPS; (2) a standardized stretching regimen targeting the hamstring muscles; and (3) a post-test measurement of the KJPS to assess changes following the stretching intervention.

**Figure 2:**
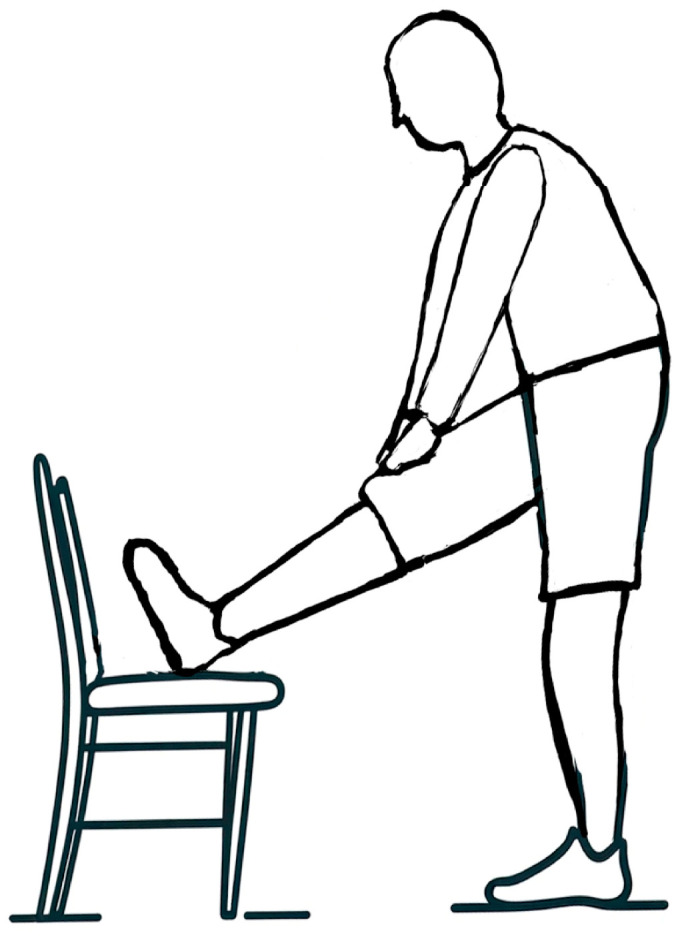
Stretching of the hamstrings.

Each participant engaged in a standardized stretching program targeting the hamstrings of their dominant leg, following both oral and written instructions. The stretching exercise was performed in a standing position, with the heel of the dominant leg placed on a chair (Figure 2). Participants were instructed to bend their trunk forward while keeping their spine upright and pushing their pelvis backward to ensure an effective stretch across the hamstrings until an intense stretch pain was felt in the back of the upper leg, which would be categorized between 7 and 8 on a pain scale from 1 to 10 (0: no stretch pain; 10: intolerable stretch pain). The participants were asked to depict the stretch sensation on a specially designed diagram. The stretching protocol consisted of self-applied passive stretching, performed three times for 30 s each. Each stretching session was followed by a 30 s rest period. After completing the stretching routine, the participants’ perception of knee joint position was assessed once again. The stretching exercise was limited to the static passive stretching method, as this method is easier for older people to perform compared to other stretching methods. In addition, the effects of the different stretching methods are comparable in terms of improving the range of motion [34,35].

**Figure 3:**
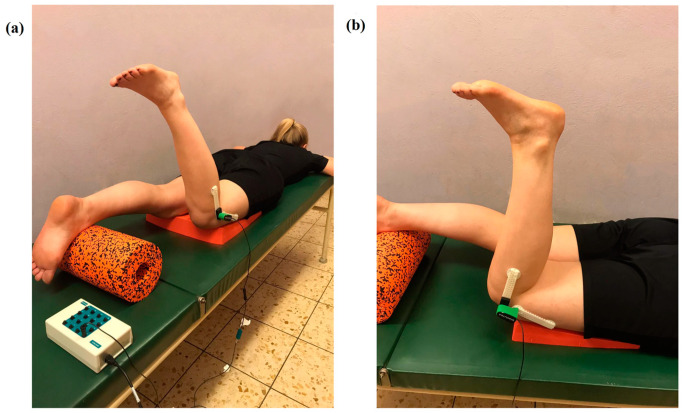
Measurement of knee joint position sense (KJPS).

The assessment of KJPS involved the evaluation of each participant’s ability to replicate the active positioning of their leg. This assessment was conducted with the participant in a prone position (Figure 3a). To facilitate the unrestricted movement of the kneecap during bending and straightening, a foam rubber pillow with a wedge shape was positioned beneath the participant’s thighs. The lower legs started on a cylindrical cushion, maintaining a standardized knee flexion of 20 degrees. During both the pre- and post-tests, participants were verbally guided by the examiner to adjust their dominant leg to a target position of 70 degrees of knee flexion and hold this position for 5 s. After returning their leg to the starting position and a brief pause of 10 s, participants were then asked to voluntarily reposition their leg to what they believed was the target angle, stating “ready” upon reaching this position. This estimated angle was held for 5 s. The accuracy of the participant’s estimation in achieving the target angle was the primary measure in assessing their KJPS.

All tests were consistently conducted in the evening, with participants being barefoot and wearing shorts to ensure uniform conditions. The same examiner provided instructions and conducted the examination for every participant to maintain consistency. Angular measurements of the knee joint were taken using an electro-goniometer with a sample rate of 100 Hz, connected to a 16-channel A/D converter (BioVision, Werheim, Germany). This goniometer was attached to the lateral aspect of the knee joint (Figure 3b). The axis of the goniometer was aligned with the knee’s flexion–extension axis, and its two lever arms were aligned with the trochanter major and malleolus landmarks for standardized positioning. Data from the angular measurements were recorded and stored using DasyLab software (Version 10, National Instruments Ireland Resources Limited, Dublin, Ireland) on a laptop computer (Fujitsu Lifebook E558, Fujitsu Limited, Tokyo, Japan).

The dependent variable recorded from the dominant leg was the difference between the target angle and the estimated angle, calculated as the absolute error (AE). Data analysis was performed using MATLAB (Version R2020b, MathWorks Inc., Natick, MA, USA).

For statistical analyses, we utilized SPSS (Version 29.0, SPSS Inc., Chicago, IL, USA). A 2 (age group) × 2 (time: pre-test vs. post-test) mixed analysis of variance (MANOVA) was employed. Any significant interaction results were subjected to simple main effect analyses, while significant main effects were followed up with post hoc analyses using Bonferroni corrections for multiple comparisons. Partial eta squared (η²) was reported as the effect size for all significant findings [36]. Data were transformed into absolute Z scores to identify statistical outliers, with a threshold value above 3 being considered as an outlier [37,38]. If an outlier was detected, the data from that participant were excluded from the statistical analysis. The significance level was set at α < 0.05. All results are presented as mean values (M) with the standard error of the mean (SEM).

## 5. Conclusions

This study expands our understanding in the field of proprioception and not only contributes to the existing knowledge base but also opens avenues for more in-depth investigations. Our findings suggest that the impact of muscle stretching on joint position sense (JPS) may differ among age groups, as we observed contrasting trends between younger and older adults. The results emphasize the need for more sophisticated research to uncover the differential effects of muscle stretching on joint position sense in different age groups, particularly between young and elderly adults. Such research is essential to develop targeted, age-appropriate rehabilitation and prevention strategies that address the specific needs of these age groups.

## Figures and Tables

**Table 1:** Study design.

Groups	Pre-Test	Treatment	Post-Test
EG_young_ (*n* = 15)	Indicating target angle (hold 5 s)	Stretching hamstrings	Indicating target angle (hold 5 s)
	Resting position (hold 10 s)	3 × 30 s (30 s rest)	Resting position (hold 10 s)
	Testing target angle (hold 5 s)		Testing target angle (hold 5 s)
EG_old_ (*n* = 15)	Indicating target angle (hold 5 s)	Stretching hamstrings	Indicating target angle (hold 5 s)
	Resting position (hold 10 s)	3 × 30 s (30 s rest)	Resting position (hold 10 s)
	Testing target angle (hold 5 s)		Testing target angle (hold 5 s)

EG_young_—experimental group of younger adults; EG_old_—experimental group of elderly adults.

## Data Availability

The data presented in this study are available upon request from the corresponding author.
